# Pseudo-outbreak of *Candida famata* due to intermittent contamination of brain heart infusion broth

**DOI:** 10.1017/ash.2026.10876

**Published:** 2026-10-09

**Authors:** Derek Larson, Paul C.F. Graf, Cameron G. Chandler, Francois Lebreton, Johnathan Amos, Daniel S. Krauth, Nanda Ramchandar, Jason W. Bennett

**Affiliations:** 1 Infectious Diseases Division, Naval Medical Center San Diego, San Diego, CA, USA; 2 Department of Medicine, Uniformed Services University, Bethesda, MD, USA; 3 Division of Microbiology, Naval Medical Center San Diego, San Diego, CA, USA; 4 Multidrug-resistant organism Repository and Surveillance Network (MRSN), Diagnostics and Countermeasures Branch, CIDR, Walter Reed Army Institute of Research, Silver Spring, MD, USA; 5 Pediatric Infectious Diseases Division, Naval Medical Center San Diego, San Diego, CA, USA; 6 Infection Prevention and Control, Naval Medical Center San Diego, San Diego, CA, USA; 7 Department of Pediatrics, Uniformed Services University, Bethesda, MD, USA

## Abstract

We describe a multidisciplinary investigation of a pseudo-outbreak of *Candida famata* involving four surgical patients at a tertiary military medical center that was traced to intermittently contaminated brain heart infusion broth. Whole-genome sequencing confirmed clonality between patient isolates and the broth.

## Introduction

*Candida famata*, historically associated with the sexual-state name *Debaryomyces hansenii*, is a rare commensal yeast with limited reports of healthcare-associated infections.^1^,^2^ Regular isolation of this yeast in clinical cultures is likely further decreased by its optimal growth between 25^°^C and 30^°^C. Misidentification of this species as *C. parapsilosis, C. (Pichia) guilliermondii, and C. lusitaniae* by biochemical methods has also been reported and may further add to its rarity.^4^,^5^ Clusters of infections have been reported that require prompt differentiation between true transmission^3^ and laboratory artifact to avoid unnecessary therapy, interventions, and operating-room closures. We report a pseudo-outbreak linked to an intermittently contaminated brain heart infusion (BHI) broth used for tissue specimen processing.

## Methods

### Case definition

A case was any patient with *C. famata* recovered from a culture processed at Naval Medical Center San Diego between December 2025 and January 2026.

### Microbiological investigation

Both primary and secondary investigations were performed in the Microbiology department, as the isolates were initially suspected to be due to contamination, though initial inspection of the specimen processing components (including the BHI broth) did not evidence a source within the laboratory. Clinical specimens from each case were cultured at 30°C and 35°C. Yeast isolates underwent identification via MALDI-TOF (Vitek MS, Knowledge Base v3.0, Biomerieux) and were sent to a commercial laboratory for antifungal sensitivities. Whole-genome sequencing was performed at the Multidrug-resistant organism Repository and Surveillance Network (MRSN) for assessment of genetic relatedness. The detailed methodology is provided as Supplemental information.

### Epidemiological investigation

Infectious Disease consultations were clinically indicated for each case, and epidemiologic links were explored. Infection Prevention reviewed operating-room logs, shared operating-room staff, surgeon schedules, and patient movements. Surgical suites were inspected for sources of contamination or lapses in maintenance or cleaning. Environmental sampling of the operating rooms was planned but discontinued upon identification of the pseudo-outbreak.

## Results

Four patients met the case definition (Table 1). All patients involved had undergone surgical procedures but otherwise had no clear epidemiologic link. Surgical procedures involved different surgeons on different days and in varied operating rooms over a 24-day period. Gram stains of original specimens showed a heavy burden of yeast with the first, third, and fourth cases and a low burden in the second case. All isolates grew only on plate media incubated at 30°C from tissue that had been ground in commercial BHI broth. Plate media incubated at 35°C showed no evidence of fungal growth. Specimens from the first two cases were replated to media and incubated at 30°C a few days after bacterial growth was detected due to the positive Gram stains for yeast, despite no fungal growth at 35°C. Subsequently, all specimens from the operating rooms were plated at 30°C and 35°C until the cause of the pseudo-outbreak was determined, leading to the recognition of the additional cases which also grew only at the lower temperature. No *C. famata* was recovered from blood, fluid, or specimens that did not require grinding or processing in BHI broth. Isolates sent to the commercial laboratory for antifungal sensitivities all failed to yield growth for testing at the recommended temperature for testing *Candida* species 35°C. All patients had an alternative infectious diagnosis to explain their clinical presentation and were successfully treated without antifungals. One patient received an empiric echinocandin until such time as the nature of the pseudo-outbreak was identified.

**Table 1.** Patient characteristics Table 1 long description.

Age (decade)	Sex	Surgical service	Procedure/Culture site	Clinical diagnosis
20s	Male	Orthopedics	Open reduction internal fixation (ORIF)	*E.coli* prosthetic infection
70s	Female	Oral maxillofacial	Jaw I and D/osteomyelitis biopsy	*S. anginosus* jaw osteomyelitis
20s	Male	Plastics	Nerve transfer reconstruction	*E.coli* infection of seroma
60s	Male	Orthopedics	Total knee arthroplasty (TKA)	Methicillin-sensitive *S. aureus* prosthetic joint infection

**Figure 1.**
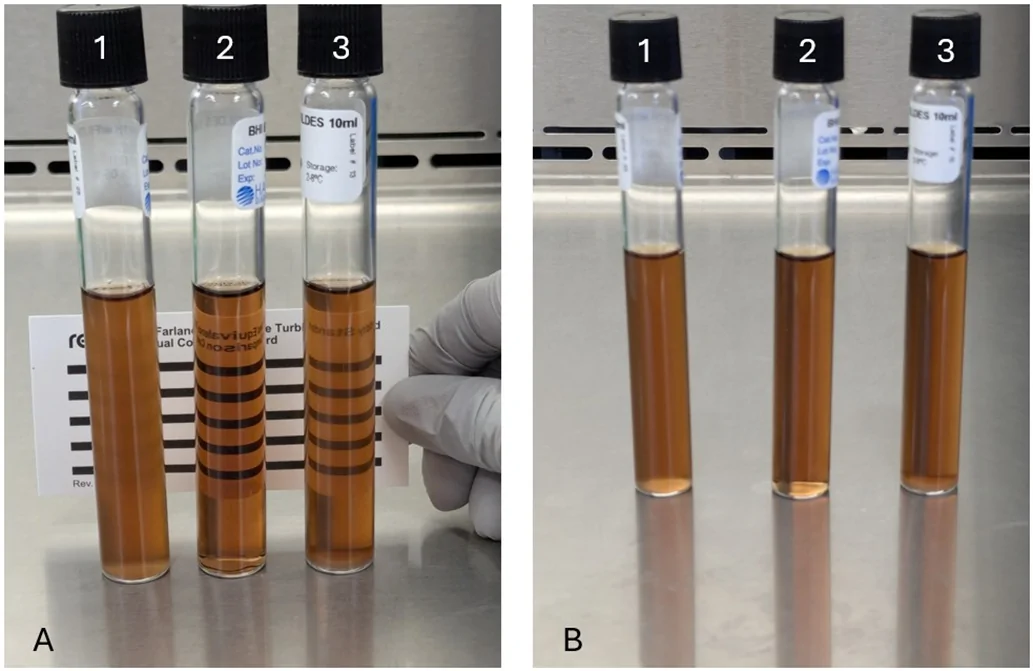
Tubes on left (1) and right (3) are contaminated, with the center tube (2) serving as an uncontaminated reference. (A) The left tube has been mixed and is visibly cloudy against an appropriate background. The contamination in the right tube has settled to the bottom and is not readily apparent without agitation of the tube. (B) The same tubes without contrast card.

Laboratory inspection revealed intermittent contamination of one BHI broth lot (Figure 1). Turbidity was not obvious on side view; a white sediment ring appeared only at the tube base that became obvious with agitation. Tubes within the same lot varied significantly in burden of contamination by sight and subculture. Yeast from uninoculated tubes from the same lot grew *C. famata* as identified by MALDI-TOF. Whole-genome sequencing confirmed clonal identity between broth contaminant and all four patient isolates. There were no insertion, deletion, or single nucleotide polymorphism differences detected between the isolates. Following resolution of the pseudo-outbreak investigation two additional tissue cultures were also discovered to be contaminated, bringing the total number of contaminated cultures to six. These last two cultures were not sent for sequencing as the outbreak investigation had been completed and the isolates were determined to be laboratory contamination. The reports for those cultures were not entered into the patients’ medical records.

## Discussion

This pseudo-outbreak resolved once the link to BHI-processed tissue specimens and intermittent contamination was identified, as well as finding no epidemiological link between the potentially affected patients. Similar *Candida* pseudo-outbreaks have been traced to laboratory media or processing steps.^6^ Here, we highlight how lower incubation temperatures may uniquely be required for growth and definitive identification of *C. famata*. Although clinical management was affected by the initial laboratory results, no patients were continued on antifungal treatments once the pseudo-outbreak was identified.

Enhanced training and a revised standard operating procedure for broth-media inspection at time of use were implemented. No further *C. famata* isolates have been recovered since sequestration of the implicated lot. The isolates from BHI broth were determined to be genetically indistinguishable from those within all four clinical samples. The manufacturer indicated that the lot in question was a small lot, they had none left in stock, and no other customer had complained about the contamination issue. The manufacturer advised the other customers of the lot-specific contamination but did not issue a full recall of the product.

## Supporting information

10.1017/ash.2026.10876.sm001 Larson et al. supplementary material Larson et al. supplementary material

## Data Availability

Genomes sequenced by the MRSN have been deposited at GenBank under Bioproject PRJNA1512451.

## Supplementary material

The supplementary material for this article can be found at https://doi.org/10.1017/ash.2026.10876.

## Disclaimer

The views expressed in this article are those of the author and do not necessarily reflect the official policy or position of Naval Medical Center San Diego, the Defense Health Agency, Department of War, U.S. Navy, or U.S. Government. All of the authors are U.S. Government employees. This work was prepared as part of their official duties. Title 17 U.S.C. § 105 provides that ‘Copyright protection under this title is not available for any work of the United States Government.’ Title 17 U.S.C. §101 defines a U.S. Government work as a work prepared by a military service member or employee of the U.S. Government as part of that person’s official duties.

The identification of specific products or scientific instrumentation is considered an integral part of the scientific endeavor and does not constitute endorsement or implied endorsement on the part of the authors, Department of War, or any component agency.

## Table 1. Long description

A table with four rows and four columns detailing patient characteristics and clinical diagnoses. The columns are labeled Age (decade), Sex, Surgical service, Procedure/Culture site, and Clinical diagnosis. Row 1: Age 20s, Sex Male, Surgical service Orthopedics, Procedure/Culture site Open reduction internal fixation (ORIF), Clinical diagnosis E.coli prosthetic infection. Row 2: Age 70s, Sex Female, Surgical service Oral maxillofacial, Procedure/Culture site Jaw landD/osteomyelitis biopsy, Clinical diagnosis S. anginosus jaw osteomyelitis. Row 3: Age 20s, Sex Male, Surgical service Plastics, Procedure/Culture site Nerve transfer reconstruction, Clinical diagnosis E.coli infection of seroma. Row 4: Age 60s, Sex Male, Surgical service Orthopedics, Procedure/Culture site Total knee arthroplasty (TKA), Clinical diagnosis Methicillin-sensitive S. aureus prosthetic joint infection.

Navigate back to Table 1..
