# Supplementary material for: Pseudo-outbreak of Candida famata due to intermittent contamination of brain heart infusion broth
Source: Antimicrob Steward Healthc Epidemiol. 2026 Oct 9;6(1):e323. doi: 10.1017/ash.2026.10876 (PMC13760387; doi:10.1017/ash.2026.10876)
Supplement: Supplementary file 1 [file S2732494X26108766sup001.docx]

**Supplemental information for:**

**Pseudo-outbreak of *Candida famata* due to Intermittent Contamination of Brain Heart Infusion Broth**

**Derek T. Larson, DO^1,6^*,* Paul C. F. Graf, PhD^2^, Cameron G. Chandler, BS^2^, Francois Lebreton, PhD^5^, Johnathan J. Amos^5^, Daniel S. Krauth, MD^1,6^, Nanda Ramchandar, MD^3,4,7^, Jason W. Bennett, MD^5^**

¹Infectious Diseases Division, Naval Medical Center San Diego, San Diego, CA, USA

²Division of Microbiology, Naval Medical Center San Diego, San Diego, CA, USA
³Pediatric Infectious Diseases Division, Naval Medical Center San Diego, San Diego, CA, USA
⁴Infection Prevention and Control, Naval Medical Center San Diego, San Diego, CA, USA
⁵Multidrug-resistant organism Repository and Surveillance Network (MRSN), Diagnostics and Countermeasures Branch, CIDR, Walter Reed Army Institute of Research, Silver Spring, MD, USA

^6^Department of Medicine, Uniformed Services University, Bethesda, MD, USA

^7^Department of Pediatrics, Uniformed Services University, Bethesda, MD, USA

*Corresponding author: Derek T. Larson, DO, FACP, FIDSA; Naval Medical Center San Diego, 34800 Bob Wilson Drive, San Diego, CA 92134, USA. Tel: +1 619-532-7475; Email: derek.larson@usuhs.edu*

**Whole genome short-read sequencing and SNP analysis.**

Whole genome sequencing (WGS) was performed for all five isolates (MRSN182586, 182587, 182588, 182589 and 182778) including four isolates from four patient samples and one isolate from the contaminated broth. For each monoculture, genomic DNA was extracted using DNeasy UltraClean Kit (QIAGEN) and aliquoted. Library construction of genomic DNA was performed with KAPA Library Quantification Kit (Roche). Libraries were quantified using the KAPA Library Quantification Kit – Illumina/Bio-Rad iCycler™ (Roche Diagnostics) on a CFX96 real-time cycler (Biorad, Hercules, CA, USA**)**. Libraries were normalized to 2nM, pooled, denatured, and diluted to 20pM. The pooled samples were further diluted to a final concentration of 16pM.

WGS was performed using a MiSeq Benchtop Sequencer (Illumina Inc., CA, USA) with MiSeq Reagent Kit v3 (600 cycle; 2 x 300bp) (Illumina, San Diego, CA, USA). Barcodes and adaptors were removed using bbduk v38.96 (Bushnell B. 2015) and reads were quality trimmed using the following parameters: ktrim="r", k="23", mink="11", hdist="1", qtrim="r", trimq="15", minlen="100". Kraken2 v2.1.2 (Wood DE. 2019) was used for initial taxonomic assignment (top hit="1", undetermined reads="<10%") and to screen for contamination (2+ genus level hits >5%="isContaminated".

*De novo* draft genome assemblies were produced using Shovill v1.1.0 (Seemann T. 2020) with coverage estimates generated using bbmap v38.96 (Bushnell B. 2015). Minimum thresholds for contig size and coverage were set at 200 bp and 49.5X+, respectively. Quality controls for the assembly were standardized with a decision tree including the following parameters: total length of contigs >1Mb; total length of contigs <1Mb over expected genome size for taxon; average read depth for each contig >=20; total length of contigs filtered for low coverage < 100kb; total length of contigs filtered for length <100kb; and number of contigs filtered for length + numbers of contigs filtered for low coverage <1,000. In cases where the Kraken2-derived taxonomic assignment was ambiguous, GTDB (Parks DH. 2022) taxonomic classification was used via the GTDB-Tk v2.4.0 using a >95% average nucleotide identity threshold for species level identification.

Genetic relatedness was first assessed by average nucleotide identity (ANI) using fastANI (Jain C. 2018) with a threshold of >99.99% ANI for identifying isolates sharing high genetic relatedness. Further, single nucleotide polymorphism (SNP) analysis was performed with Snippy v.4.4.5 (Seemann T. 2020) using error corrected [Pilon v1.23] (Walker BJ. 2014) and the draft assembly of the earliest isolate (by date) was chosen as reference. The core alignment was filtered for recombination using Gubbins v2.3.156 (Croucher NJ.2015) and the SNP counts were obtained using SNP-dist (Seemann T. 2018) with a threshold of <10 SNPs for clonal isolates.

**References**

Bushnell B. 2015. BBMap: short read aligner for DNA/RNAseq, and other bioinformatic tools. <https://github.com/BioInfoTools/BBMap>.

Wood DE, Lu J, Langmead B. Improved metagenomic analysis with Kraken 2. Genome Biol. 2019 Nov 28;20(1):257. doi: 10.1186/s13059-019-1891-0. PMID: 31779668; PMCID.

Seemann T. 2020. Shovill: assemble bacterial isolate genomes from Illumina paired-end reads. <https://github.com/tseemann/shovill>.

Parks DH, Chuvochina M, Rinke C, Mussig AJ, Chaumeil PA, Hugenholtz P. 2022. GTDB: an ongoing census of bacterial and archaeal diversity through a phylogenetically consistent, rank normalized and complete genome-based taxonomy. Nucleic Acids Res 50:D785-D794.

Seemann T. 2020. Snippy: rapid haploid variant calling and core genome alignment. <https://github.com/tseemann/snippy>.

Walker BJ, Abeel T, Shea T, Priest M, Abouelliel A, Sakthikumar S, Cuomo CA, Zeng Q, Wortman J, Young SK, Earl AM. 2014. Pilon: an integrated tool for comprehensive microbial variant detection and genome assembly improvement. PLoS One 9:e112963.

Jain C, Rodriguez-R LM, Phillippy AM, Konstantinidis KT, Aluru S. High throughput ANI analysis of 90K prokaryotic genomes reveals clear species boundaries. Nat Commun. 2018 Nov 30;9(1):5114. doi: 10.1038/s41467-018-07641-9. PMID: 30504855; PMCID: PMC6269478.

Croucher NJ, Page AJ, Connor TR, Delaney AJ, Keane JA, Bentley SD, Parkhill J, Harris SR. 2015. Rapid phylogenetic analysis of large samples of recombinant bacterial whole genome sequences using Gubbins. Nucleic Acids Res 43:e15.
Seemann, T. (2018). Source code for snp-dists software (Version 0.6.2) [Computer software]. Zenodo. <https://doi.org/10.5281/zenodo.1411986>
